# Supplementary figures and images for: Functional and evolutionary perspectives on gill structures of an obligate air-breathing, aquatic snail
Source: PeerJ. 2019 Jul 31;7:e7342. doi: 10.7717/peerj.7342 (PMC6679647; doi:10.7717/peerj.7342)

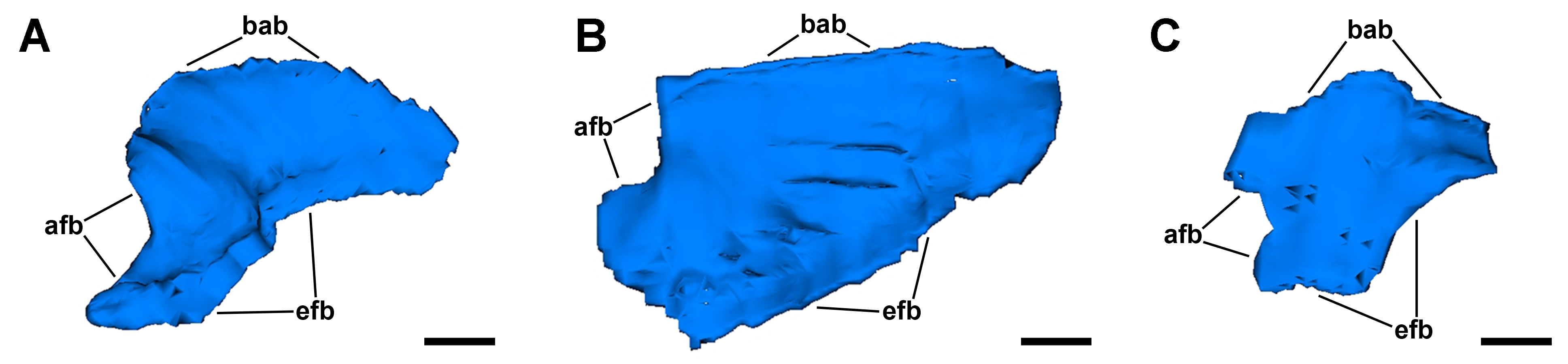

Supplement: Supplemental Information 1 — (A) Anterior leaflet. (B) Intermediate leaflet. (C) Posterior leaflet. Scale bars represent 500 μm. Abbreviations: afb, afferent border; bab, basal border; efb, efferent border. [file peerj-07-7342-s001.png]

**A**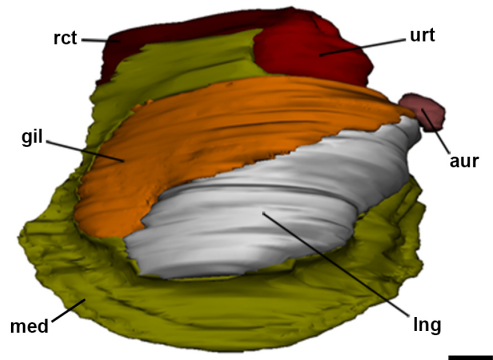**B**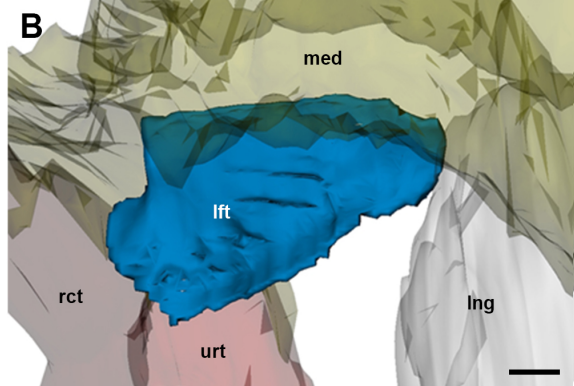**C**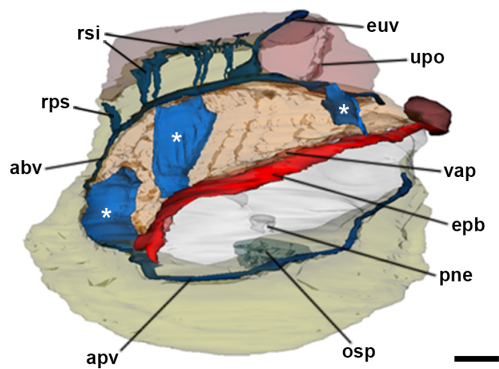**D**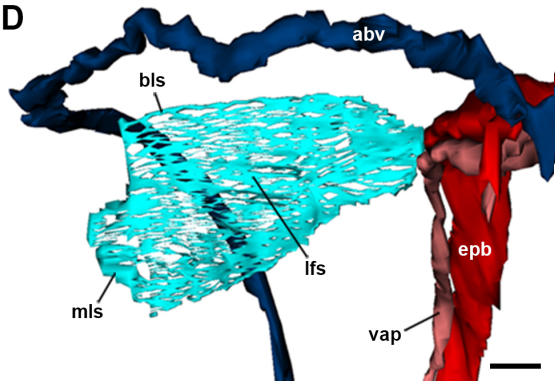

Supplement: Supplemental Information 2 — Left click on the figure to activate the 3D model using Adobe Acrobat Reader. Left click and drag to rotate, right click and drag to zoom-in/out. Structures can be shown/hidden by selecting the pre-defined views from the dropdown menu in the view panel. The 3D PDF was generated using the 3D tool of Adobe Acrobat 9 Pro Extended by importing VRML 2.0 files from Reconstruct v.1.1.0.0. [file peerj-07-7342-s002.pdf]
