# Supplementary material for: Functional and evolutionary perspectives on gill structures of an obligate air-breathing, aquatic snail
Source: PeerJ. 2019 Jul 31;7:e7342. doi: 10.7717/peerj.7342 (PMC6679647; doi:10.7717/peerj.7342)
Supplement: Supplemental Information 3 [file peerj-07-7342-s003.docx]

| Andrews (1965) | | This paper | | Comments |
| --- | --- | --- | --- | --- |
| Abbreviation | **Structure** | **Abbreviation** | **Structure** |  |
| akc | anterior chamber of kidney | urt | ureter | — |
| apv | afferent pulmonary vein;  dorsal afferent pulmonary vein | apv | afferent pulmonary vessel | This vessel is not directly connected with the efferent pulmobranchial vessel or with the ventral afferent pulmonary vessel, as Figure 6b in Andrews (1965) suggests |
| asl | afferent sinus of ctenidial leaflet | mls | marginal leaflet sinus | There is a single marginal sinus |
| cta | ctenidial artery | — | — | Not found in our study |
| ebv; epv | efferent branchial vein; efferent ctenidial and pulmonary vein | epb | efferent pulmobranchial vessel | This vessel is connected at its anterior end with the ventral afferent pulmonary vessel. This seems to have been overlooked by Andrews (1965); see her Figure 6b |
| erv | efferent renal vein | euv | efferent ureteral vessel | — |
| esl | efferent sinus of ctenidial leaflet | mls | marginal leaflet sinus | See the entry for ‘asl’ |
| ko | opening of kidney into mantle cavity | npo | nephropore | — |
| pkc | posterior chamber of kidney | kid | kidney | — |
| spv | superficial pallial vein | — | — | Not found in our study |
| sts | superficial transverse channels; transverse channels  through mantle skirt | bls | basal leaflet sinus | — |
| tsl | transverse sinuses in  ctenidial leaflets | lfs | laminar leaflet sinus | There are not transverse sinuses but a single laminar sinus interrupted by trabeculae |
| vap | ventral afferent pulmonary vein | vap | ventral afferent pulmonary vessel | See the entry for ‘ebv’ |
